# Validation of the alcohol use disorders identification test in a Danish hospital setting

**DOI:** 10.1186/s13011-025-00638-w

**Published:** 2025-02-14

**Authors:** Peter Næsborg Schøler, Max Benjamin Andersen, Kjeld Andersen, Ulrik Becker, Maja Thiele, Anette Søgaard Nielsen

**Affiliations:** 1 Unit for Clinical Alcohol Research, Research Unit of Psychiatry, Department of Clinical Research, University of Southern Denmark, Odense, Denmark; 2 Department of Psychiatry Odense, Mental Health Services Region of Southern Denmark, Odense, Denmark; 3 Brain Research - Inter-Disciplinary Guided Excellence, BRIDGE, University of Southern Denmark, Odense, Denmark; 4 National Institute of Public Health, University of Southern Denmark, Odense, Denmark; 5 Center for Liver Research, Department of Gastroenterology and Hepatology, Institute of Clinical Research, Faculty of Health, Odense University Hospital, University of Southern Denmark, Odense, Denmark

**Keywords:** Alcohol Use Disorder, Alcohol-related disorders, Clinical decision-making, Validation study, Alcohol Use disorders Identification Test, Screening.

## Abstract

**Background:**

Early identification of potential alcohol-problems is central for timely intervention and treatment referral. The Alcohol Use Disorders Identification Test (AUDIT) and AUDIT-Consumption (AUDIT-C) serve as globally recognized and validated screening tools for this purpose. We aimed to evaluate the diagnostic validity of internationally recommended AUDIT cut-off scores ≥ 8, ≥16, ≥ 20, and AUDIT-C cut-off scores ≥ 4, ≥5 using the Danish language versions of questionnaires in a hospital setting.

**Methods:**

Questionnaire data were collected from 2/15/2023, to 4/27/2023 at the Department of Gastroenterology and Hepatology, Odense University Hospital, Denmark. We tested the World Health Organization’s recommended AUDIT cut-offs: ≥8 for hazardous use, ≥ 16 suggestive of dependence, ≥ 20 high likelihood of dependence, along with AUDIT-C ≥ 4 and ≥ 5 using the following reference standard: Danish low-risk drinking guidelines (≤ 10 standard drinks/week) for hazardous use and self-reported ICD-10 alcohol dependence criteria for alcohol dependence. Analyses included ROC curves, AUC, sensitivity, specificity, predictive values, and agreement.

**Results:**

Three hundred patients participated, mean age 52 years (SD 17.4, median 54) and 51.3% males. Mean AUDIT score was 4.5 (SD 5.8, median 3) with fourteen (4.7%) meeting at least three self-reported ICD-10 criteria for alcohol dependence. The prevalence of hazardous use was 10.7%. AUDIT ≥ 8 exhibited a sensitivity of 56% (95% CI 40.6–73.6) and specificity 91% (95% CI 87.8–94.5) for detecting hazardous use. Against at least three self-reported ICD-10 criteria for alcohol dependence, AUDIT cut-off ≥ 16 showed a sensitivity of 85% (95% CI 66.1–98.2) with 97% specificity (95% CI 96.0-99.2), while cut-off ≥ 20 had a sensitivity of 71% (95% CI 49.2–91.6) with 99% specificity (95% CI 98.1–99.9). The AUDIT-C cut-offs ≥ 4 and ≥ 5 exhibited low positive predictive values in detecting hazardous use (30.8% for ≥ 4 and 36.8% for ≥ 5) and dependence (13.5% for ≥ 4 and 18.4% for ≥ 5) and demonstrated a specificity ranging from 68.5 to 82.1% with negative predictive values from 98.2 to 100%.

**Conclusion:**

In Danish gastroenterology and hepatology departments, the AUDIT and AUDIT-C may be used to identify patients who are unlikely to have an alcohol problem, while positive screen results should be carefully considered and followed by more exhaustive assessment.

**Supplementary Information:**

The online version contains supplementary material available at 10.1186/s13011-025-00638-w.

## Background

Three million annual deaths, approximately 5.3% of total global deaths, are attributable to alcohol use [1]. Alcohol use disorder (AUD) has one of the largest treatment gaps of any health condition with only 14–17% of individuals with AUD receiving alcohol treatment [2]. Identifying individuals with hazardous alcohol use or AUD through screening is crucial for raising awareness of the potential harms of alcohol and facilitating treatment or referral [3].

The Alcohol Use Disorders Identification Test (AUDIT), developed in 1993 as a World Health Organization collaborative project [4], stands as one of the most widely used alcohol screening instruments. It is a ten-item self-report questionnaire originally developed for adult primary care patients [5] and has undergone validation across various languages, settings, and population samples [6–9]. The AUDIT is often described as a tool for identifying “hazardous or harmful” alcohol consumption and these terms are often used interchangeably [10–14] although there are distinct differences. Hazardous use represents significant risk of future harm without current or demonstrable harm to one’s health [15] while harmful use involves the presence of demonstrable harm caused by alcohol to the person’s physical or mental health [15]. Alcohol dependence syndrome is defined as a chronic, relapsing disease characterized by physical and mental dependence on alcohol often associated with severe symptoms [16]. Recommended cut-off scores on the AUDIT are ≥ 8 for hazardous use, ≥ 16 suggestive of alcohol dependence, and ≥ 20 indicating high likelihood of alcohol dependence [5, 12]. AUDIT scores ≥ 8 have been found associated with increased all-cause mortality [13] and the psychometric properties of the test are generally high [6].

The AUDIT-Consumption (AUDIT-C), a shorter version of the AUDIT focusing on the AUDIT’s first three questions on the quantity of the alcohol intake, has also demonstrated good validity and reliability in various populations and settings for identifying hazardous use and dependence [6, 17]. The recommended AUDIT-C cut-off scores are typically 5 or 6 for men and 3 or 4 for women, depending on the specific context and goals of the screening [6, 11, 18]. In Denmark, there is currently no validated gold standard for self-reported or questionnaire-based screening for alcohol problems. This extends to the AUDIT and AUDIT-C, despite their widespread utilization in both clinical practice and research. Notably, the AUDIT is recommended by the Danish College of General Practitioners and the Danish Health Authorities [19].

This study aimed at investigating the psychometric properties of the Danish versions of the AUDIT and AUDIT-C questionnaire in a Danish hospital setting. Specifically, we aimed to test the diagnostic validity of internationally recommended cut-off scores of AUDIT and AUDIT-C to identify hazardous use and the likelihood of alcohol dependence in a patient population.

## Methods and materials

### Design

Cross sectional study, performed at Department of Gastroenterology, Odense University Hospital, Odense, Denmark, from February 15, 2023, to April 27, 2023.

Self-reported AUDIT and AUDIT-C scores were tested against two standards which functioned as reference standards rather than true gold standard comparisons [20]. The first standard was an alcohol intake of 10 standard drinks (12 g of alcohol) or more assessed by the Timeline Follow-Back (TLFB) one-week version [21] as the reference standard for hazardous alcohol consumption in accordance with the Danish National Board of Health’s recommendations [22]. The second standard was self-reported ICD-10 criteria for alcohol dependence based on the ICD-10 clinical descriptions and diagnostic guidelines [16] as reference standard for dependence.

This study followed the Standards for Reporting Diagnostic Accuracy (STARD) statement [23]. The STARD checklist is presented in Supplementary Material 1.

### Study population

Eligible participants included in- and outpatients ≥ 18 years of age, proficient in both written and spoken Danish, and admitted to the Department of Gastroenterology, Odense University Hospital, Odense, Denmark. We excluded individuals unable to complete the questionnaire due to illness or cognitive impairment and those unable to comprehend written and spoken Danish. We selected the department because previous studies showed a higher prevalence of hazardous alcohol use and dependence among its patients compared to the general Danish population [24, 25], aligning with trends observed in hospitalized patients in both Denmark [26] and Europe [27], concerning gender, age, and alcohol consumption patterns. The sample comprised new referrals, e.g., from primary care or other hospital wards, and ongoing patients in both the in- and outpatient clinic. While all were, by definition, treatment-seeking, their primary concerns were not necessarily alcohol-related. Based on the prevalence estimates presented above, we selected a minimum sample size of 300 participants to ensure adequate power (> 80%) with a 5% margin of error [28].

### Procedure

Participants were approached using convenience sampling [29] at the hospital-based specialist outpatient clinic and the inpatient ward located on the floor above the outpatient clinic. Two research assistants, unaffiliated with the department staff, approached patients in the in- and outpatient clinics. They identified themselves as representatives from the Research Unit of Psychiatry Odense, emphasizing participant anonymity and the study’s separation from the patients’ treatment. Wearing distinctly different clothes to differentiate themselves from the department staff, the assistants explained the purpose of the study, informed that responses would not be shared with department staff, and clarified that participation would not affect other activities, treatment, or care in the department. They then addressed any patient questions. Participants completed the questionnaire electronically on a tablet, with responses directly uploaded to a secure Research Electronic Data Capture (REDCap) [30] database provided by Odense Patient data Explorative Network (OPEN) [31]. Finally, the assistants gave each participants a thank-you card for their involvement in the study. The back of the card included contact details for the local alcohol specialist treatment facility, along with a brief note encouraging participants to reach out if the study or any questionnaires had raised concerns or questions about their alcohol use.

### Questionnaires

The patient questionnaire included questions on age, gender (man, woman, other, do not wish to inform), and the following four assessment tools.

*The Alcohol Use Disorders Identification Test (AUDIT)* [5], a ten-item self-report questionnaire on alcohol consumption, alcohol-related harm, and alcohol dependence ranging from 0 to 40 point. Questions 1–8 are scored from 0 to 4 points on a Likert scale, questions 9 and 10 have three responses scoring 0, 2, or 4 points. We used the Danish version of the AUDIT as it is provided by the Danish Health Authorities and Danish Collage of General Practitioners [19]. However, no formally validated Danish translation of the AUDIT exists for reference.

*The International Classification of Diseases (ICD-10)* [16] criteria for alcohol dependence syndrome (F10.2) framed as questions, i.e. not diagnostic. According to the ICD-10 criteria for alcohol dependence, three or more of six symptoms should be present for a minimum of one month, or repeatedly presented within a one-year period. The patient questionnaire included the six ICD-10 criteria framed as self-report questions, e.g. “do you experience a strong desire or sense of compulsion (craving) to drink alcohol?” (criteria 1) adapted from the Danish translation of the ICD-10 criteria for Danish national guidelines [19]. Supplementary material 3 features the adapted ICD-10 criteria as self-report questions (in Danish).

*The Timeline Follow Back (TLFB)* [21] one-week version. The participant fills in last week’s alcohol consumption in a calendar format. The TLFB one-week version has been applied successfully through questionnaires and in online format [32–34].

### Statistics

**Fig. 1:**
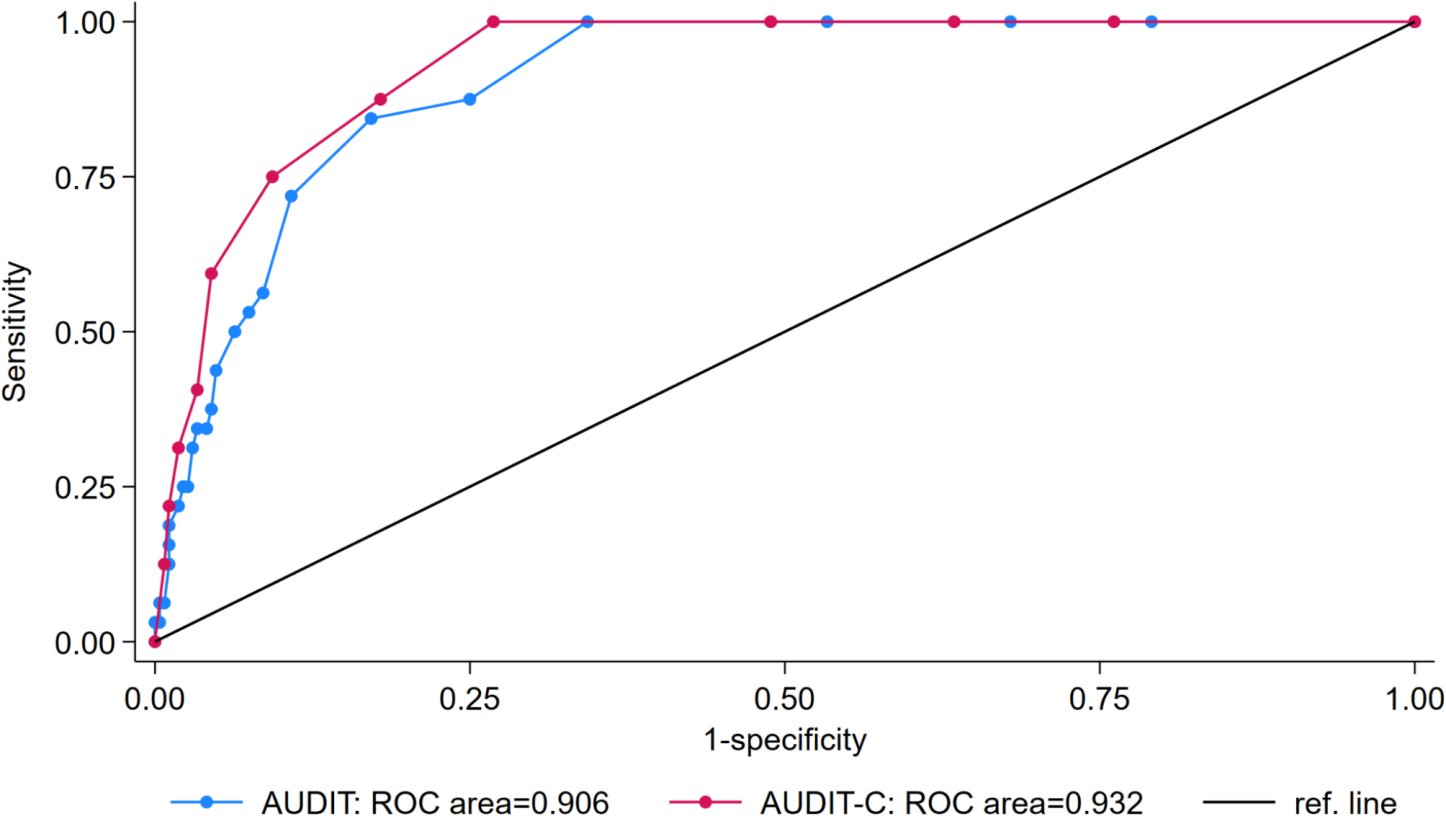
Receiver operator curve of The Alcohol Use Disorder Identification Test (AUDIT) and The Alcohol Use Disorder Identification Test-Consumption (AUDIT-C) versus hazardous alcohol use (Weekly alcohol intake > 10 drinks) as reference standard, all participants, *n* = 300

**Fig. 2:**
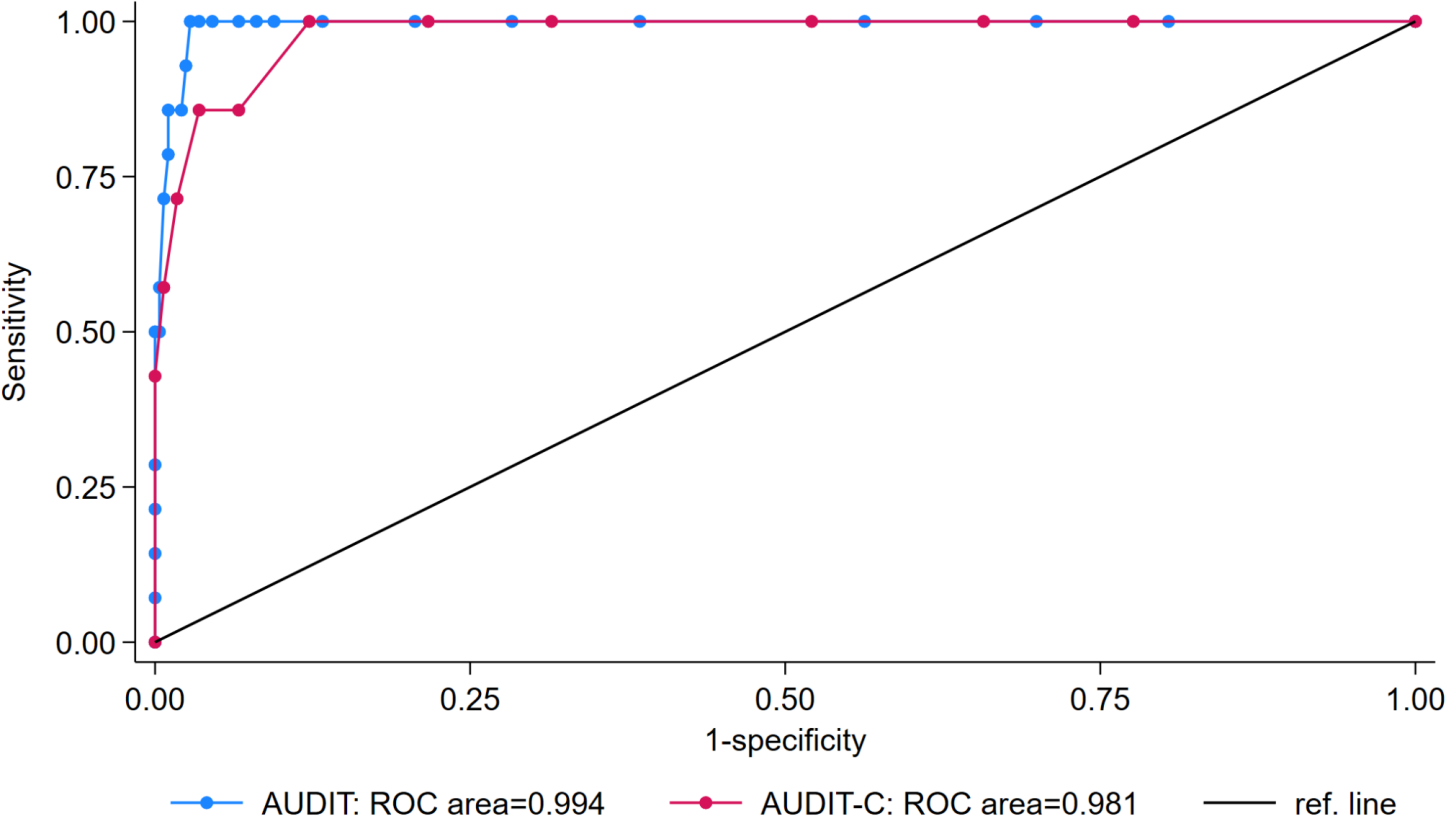
Receiver operator curve of the alcohol use disorder identification test (AUDIT) and the alcohol use disorder identification test-consumption (AUDIT-C) versus the international classification of diseases 10th revision (ICD-10) dependence (≥ 3 self-reported ICD-10 symptoms) as reference standard, all participants, *n* = 300. Notes: ROC, Receiver Operating Characteristics

We conducted internal consistency analysis of the AUDIT and AUDIT-C using Cronbach’s alpha test and performed receiver operator characteristics (ROC) curve analysis. Analysis included internationally recommended AUDIT scores ≥ 8 (indicating hazardous use), ≥ 16 (suggestive of dependence), and ≥ 20 (high likelihood of dependence), as well as AUDIT-C scores ≥ 4 and ≥ 5. We compared cut-offs to the ICD-10 criteria for alcohol dependence syndrome (F10.2) with ≥ 3 symptoms as reference standard for dependence. Additionally, we evaluated adherence to Danish health authorities’ national recommendations for maximum weekly alcohol intake of 10 standard drinks, measured by TLFB, as reference standard for hazardous use. We analyzed the entire sample and subsequently stratified by sex. We assessed the area under the ROC curve (AUC) and diagnostic properties (sensitivity, specificity, positive predictive value (PPV), negative predictive value (NPV), and agreement) of the AUDIT and AUDIT-C cut-off scores based on the self-reported ICD-10 criteria and national guidelines for hazardous use. We calculated both AUC based on continuous AUDIT scores (Figs. 1 and 2) and computed separate AUCs for each cut-off by dichotomizing AUDIT scores according to the three cut-off values 8, 16, and 20 (Tables 1 and 2) [35]. For skewed data, mean, standard deviation, median and range was calculated. This included age, alcohol intake, AUDIT score, and AUDIT-C score (Table 3). Finally, we estimated optimal cut-off point for AUDIT and AUDIT-C according to the two reference standards using Youden’s Index [36]. We used STATA version 17 for the analyses.

**Table 1:** Participant characteristics *N* = 300

	All participants *N* = 300	Fulfills ≥ 3 self-reported ICD-10 criteria for alcohol dependence syndrome *N* = 14	Does not fulfill the ICD-10 criteria for alcohol dependence *N* = 286	Significance level of difference, *p*-value
Female gender, *n* (%)	145 (48.3)	4 (28.6)	141 (49.3)	0.130
Age in years, mean (SD) *median [range]*	52.0 (17.4) *54 [18*,* 86]*	54.1 (14.0) *55.5 [19*,* 73]*	51.9 (17.6) *53.5 [18*,* 86]*	0.679
Alcohol intake last week^a^ in standard drinks, mean (SD) *median [range]*	4.1 (8.7) *0 [0*,* 60]*	16.6 (22.3) *7.5 [0*,* 60]*	3.5 (7.0) *0 [0*,* 46]*	0.014
Alcohol intake last week^a^, > 10 standard drinks (%) (hazardous use)	32 (10.7)	6 (42.9)	26 (9.1)	< 0.001
AUDIT score^b^, mean (SD) *median [range]*	4.5 (5.8) *3 [0*,* 38]*	24.1 (7.2) *25 [13*,* 38]*	3.5 (3.6) *3 [0*,* 25]*	< 0.001
AUDIT score^b^ ≥8 (%) (hazardous use)	41 (13.7)	14 (100)	27 (9.4)	< 0.001
AUDIT score^b^ ≥16 (%) (suggestive of dependence)	18 (6.0)	N/A	N/A	< 0.001
AUDIT score^b^ ≥20 (%) (high likelihood of dependence)	12 (4.0)	N/A	< 3	< 0.001
AUDIT-C score^c^, mean (SD) *median [range]*	3.1 (2.7) *3 [0*,* 12]*	9.9 (2.2) *10 [6*,* 12]*	2.7 (2.3) *3 [0*,* 10]*	< 0.001
AUDIT-C score^c^ ≥4 (%)	104 (34.7)	14 (100)	90 (31.5)	< 0.001
AUDIT-C score^c^ ≥5 (%)	76 (25.3)	14 (100)	62 (21.7)	< 0.001

Notes: SD, Standard Deviation. AUDIT, Alcohol Use Disorder Identification Test. AUDIT-C, Alcohol Use Disorder Identification Test-Consumption. N/A: Results cannot be shown due to European general data protection regulations (at least one cell with < 3 observations). ^a^Alcohol intake during the last week, recorded per day by means of The Timeline Follow Back one week version and summarized. Alcohol intake is measured as number of standard drinks (12 g of pure alcohol). ^b^AUDIT score range 0–40. Higher score indicating higher levels of alcohol problems. World Health Organization cut-off values for AUDIT scores: hazardous use (AUDIT ≥ 8), suggestive of dependence (AUDIT ≥ 16), high likelihood of dependence (AUDIT ≥ 20). ^c^AUDIT-C score range 0–12. Higher score indicating higher alcohol consumption. Standard drink is 12 g of alcohol

**Table 2:** Diagnostic properties of the AUDIT cut-off values 8, 16, and 20. *N* = 300

	AUC	Sensitivity	Specificity	PPV	NPV	Agreement
	proportion (95%-CI)	% (95%-CI)	% (95%-CI)	% (95%-CI)	% (95%-CI)	% (95%-CI)
**Dependence ** **(≥ 3 self-reported ICD-10 criteria)**						
*AUDIT ≥ 20*	0.85 (0.73, 0.98)	71.4 (49.2, 91.6)	99.3 (98.1, 99.9)	83.3 (61.5, 97.9)	98.6 (97.0, 99.6)	98.0 (96.2, 99.3)
*AUDIT ≥ 16*	0.92 (0.82, 1.00)	85.7 (66.1, 98.2)	97.9 (96.0, 99.2)	66.7 (46.5, 86.7)	99.3 (98.0, 99.9)	97.3 (95.3, 98.8)
**Hazardous use (Weekly use > 10 drinks/week)**						
*AUDIT ≥ 8*	0.74 (0.65, 0.83)	56.3 (40.6, 73.6)	91.4 (87.8, 94.5)	43.9 (30.7, 60.3)	94.6 (91.6, 97.0)	87.7 (83.8, 91.2)

Notes: Diagnostic properties for AUDIT ≥ 20 (high likelihood of dependence) according to at least three ICD-10 criteria for alcohol dependence syndrome, for AUDIT ≥ 16 (suggestive of dependence) according to at least three ICD-10 criteria for alcohol dependence syndrome, and AUDIT ≥ 8 (hazardous use) according to weekly alcohol intake > 10 standard drinks/week. AUDIT, The Alcohol Use Disorder Identification Test. ICD-10, International Classification of Diseases 10th revision. AUC, area under the ROC curve, dichotomized for the respective cut-offs. ROC, receiver operator characteristics. PPV, positive predictive value. NPV, negative predictive value. CI, Clopper-Pearson binomial confidence interval. Agreement, the degree to which the test matches the results of the reference standard (ICD-10 criteria for alcohol dependence syndrome ≥ 3 and Weekly use > 10 drinks/week). One standard drink = 12 g of alcohol

**Table 3:** Diagnostic properties of the AUDIT-C for cutoff values 4 and 5. *N* = 300

	AUC	Sensitivity	Specificity	PPV	NPV	Agreement
	proportion (95%-CI)	% (95%-CI)	% (95%-CI)	% (95%-CI)	% (95%-CI)	% (95%-CI)
**Dependence (≥ 3 self-reported ICD-10 criteria)**						
*AUDIT-C ≥ 4*	0.84 (0.82, 0.87)	100 (N/A)	68.5 (63.2, 73.9)	13.5 (8.3, 21.6)	100 (N/A)	70.0 (64.8, 75.1)
*AUDIT-C ≥ 5*	0.89 (0.87, 0.92)	100 (N/A)	78.3 (73.5, 83.0)	18.4 (11.5, 29.0)	100 (N/A)	79.3 (74.7, 83.8)
**Hazardous use (Weekly use > 10 drinks/week)**						
*AUDIT-C ≥ 4*	0.87 (0.84, 0.89)	100 (N/A)	73.1 (67.8, 78.4)	30.8 (23.0, 40.6)	100 (N/A)	76.1 (71.1, 80.7)
*AUDIT-C ≥ 5*	0.85 (0.79, 0.87)	87.5 (75.0, 96.5)	82.1 (77.4, 86.5)	36.8 (27.3, 48.7)	98.2 (96.1, 99.5)	82.3 (78.3, 86.8)

Notes. Diagnostic properties of the AUDIT-C cut-off values 4 and 5 according to at least three ICD-10 criteria for alcohol dependence syndrome, and according to weekly alcohol intake > 10 standard drinks/week. AUDIT-C, Alcohol Use Disorder Identification Test-Consumption. ICD-10, International Classification of Diseases 10th revision. AUC, area under the ROC curve, dichotomized for the respective cut-offs. ROC, receiver operator characteristics, PPV, positive predictive value, NPV, negative predictive value, CI, Clopper-Pearson binomial confidence interval, N/A, not applicable. Agreement, the degree to which the test matches the results of the reference standard (ICD-10 criteria for alcohol dependence syndrome ≥ 3 and Weekly use > 10 drinks/week). One standard drink = 12 g of alcohol

## Results

A total of 317 patients participated, with seventeen incomplete entries, resulting in 300 observations for analysis. The mean age was 52 years (SD = 17.4) and the majority were men (51.3%). Fourteen (4.7%) met ≥ 3 self-reported ICD-10 criteria for alcohol dependence. Mean alcohol consumption and mean AUDIT and AUDIT-C scores were higher among participants meeting ≥ 3 self-reported ICD-10 criteria compared to others (Table 3).

The internal consistency of the AUDIT and the AUDIT-C was high, with Cronbach’s alpha at α = 0.88 in both cases.

The AUDIT exhibited low sensitivity in detecting hazardous use (cut-off ≥ 8), with a sensitivity of 56% and PPV of 44%. However, it demonstrated a specificity for hazardous use of 91% with an NPV of 95% (Table 1). Figure 1 presents the ROC curves for the AUDIT and the AUDIT-C versus a weekly alcohol intake > 10 standard drinks as reference standard for hazardous use.

The AUDIT cut-off ≥ 16, suggestive of dependence, showed a sensitivity of 86% but with a low PPV of 67%. Again, the specificity was high at 98% with a high NPV at 99%. The sensitivity for detecting a high likelihood of dependence (cut-off ≥ 20) was 71% with a PPV of 83%, while the specificity was 99%, with a NPV of 99% (Table 1). Figure 2 presents the ROC curve and AUC for the AUDIT and AUDIT-C versus ≥ 3 self-reported ICD-10 criteria for alcohol dependence syndrome.

We also evaluated the AUDIT-C cut-offs ≥ 4 and ≥ 5 for detecting hazardous use (weekly consumption > 10 standard drinks), and for detecting dependence (≥ 3 ICD-criteria for alcohol dependence). The AUDIT-C cut-offs ≥ 4 and ≥ 5 demonstrated high sensitivity in detecting both hazardous use and dependence, but PPV values were low, ranging from 14 to 37% (Table 2). Its specificity for hazardous use and dependence were lower than its sensitivity, ranging from 69 to 82% but the NPVs were high (98–100%). An AUDIT-C score ≥ 4 had a specificity for hazardous use of 73% (NPV 100%) and 69% for dependence (NPV 100%). The specificity increased with a cut-off of ≥ 5, demonstrating 82% specificity for hazardous use (NPV 98%), and 78% specificity for dependence (NPV 100%).

For the self-reported ICD-10 criteria of alcohol dependence, the Youden’s index estimates for optimal cut-off were AUDIT ≥ 13 and AUDIT-C ≥ 6. For the reference standard of weekly use > 10 drinks/week (hazardous use), Youden’s index estimates for optimal cut-offs were AUDIT ≥ 6 and AUDIT-C ≥ 4. Sensitivity, specificity, and agreement for all AUDIT and AUDIT-C cut-off values including estimates for optimal cut-off values according to the reference standards using Youden’s Index [36], are presented in Supplementary Material 2.

The stratified ROC curve analyses for the AUDIT and AUDIT-C showed no sex differences (data not shown). However, the sample included limited data for women with higher AUDIT and AUDIT-C scores.

## Discussion

This study investigated the diagnostic properties of the Alcohol Use Disorders Identification Test (AUDIT) and the AUDIT-Consumption (AUDIT-C) in a patient sample from a Danish department of gastroenterology and hepatology. Both scales demonstrated high internal consistency with high specificity and high negative predictive values (NPVs) against self-reported reference standards of hazardous use and dependence.

The AUDIT exhibited higher sensitivity for dependence than for detecting hazardous use. The observed study sample was similar to other in-hospital population in Denmark regarding age and gender [24, 26] but our findings diverge from a meta-analysis which found that the AUDIT performs poorly in identifying AUD when the prevalence is low [14]. However, it is crucial to note that any test’s performance and PPV decrease with lower population prevalence of the condition [37, 38]. Despite a lower-than-expected prevalence of “high likelihood of dependence” i.e. an AUDIT score > 20, in our study (4.7%), the prevalence was not much different than in the Danish general population [39]. For context, a recent representative population surveys indicate that 4% of males and 0.8% of females in Denmark consume more than 30 standard drinks per week, while 15.7% exceed the national recommended limit of 10 drinks per week [40] (10.7% in our study sample).

The AUDIT-C cut-offs of ≥ 4 and ≥ 5 exhibited high sensitivity but low PPV according to the self-reported ICD-10 criteria for dependence and Danish national recommendations for hazardous use. Reinert et al. observed that the AUDIT-C’s sensitivity generally seems to be higher for dependence than for lower intensity alcohol problems and argued this might be due to the demarcated nature of dependence [7]. We were unable to evaluate whether the AUDIT-C’s sensitivity was higher for dependence as the prevalence of “high likelihood of dependence” was low in the sample.

We anticipated a larger variation in alcohol consumption levels and higher AUDIT scores overall in the study sample based on the estimated prevalence of harmful alcohol use in Denmark (18% of men and 9% of women) [40, 41] and studies reporting higher AUDIT scores among in-hospital patients compared to the general population [27]. Additionally, a prior study conducted at the same department as our study took place, found high AUDIT scores among inpatients [24]. However, contrary to our expectations, we did not observe significant variation in AUDIT scores, and there were few cases of AUDIT scores ≥ 20 (4%). These findings could be attributed to the data collection setting, which included a hospital-based specialist outpatient clinic and an inpatient ward primarily treating patients with liver disease. Patients in this setting may receive intensive ongoing treatment or attend follow-up appointments post-treatment, potentially leading to reduced alcohol consumption due to factors such as the burden of their disease or the nature of their treatment. Patients with alcohol-related liver disease, who often participated in this study, may be more motivated to reduce or quit drinking compared to the general population. Equally, other patient groups within the gastroenterology department, such as those with inflammatory bowel disease, typically consume less alcohol than their healthy peers, a phenomenon known as the “sick-quitter effect” [42].

Despite these unexpected findings, both the AUDIT and AUDIT-C demonstrated high specificity and NPVs, which may help clinicians to identify patients unlikely to have an alcohol problem with high certainty. However, caution is warranted, especially for the AUDIT-C when interpreting positive screen scores. One application of these findings is that the AUDIT-C could be utilized as an initial screening tool to help the clinician identify patients unlikely to have an alcohol problem, but moving on to a full AUDIT assessment if the patient scores above a certain cut-off as recommended in other studies [43]. However, as our data contained few observations on individuals with higher AUDIT scores, especially regarding women and across different age groups, we were unable to discern meaningful gender-specific or age-specific cut-offs.

We also estimated Youden’s index optimal cut-off scores for the AUDIT and AUDIT-C based on our two reference standards. For ≥ 3 self-reported ICD-10 symptoms, the optimal scores were ≥ 13 for AUDIT and ≥ 6 for AUDIT-C, while for weekly intake > 10 drinks, they were ≥ 6 and ≥ 4 respectively (see Supplementary Material 2). However, our primary objective was to evaluate the diagnostic validity of internationally recommended AUDIT cut-off scores, given their current use in Danish clinical practice without local validation. This evaluation is essential for clinicians to determine whether they can reliably apply these scores in their practice. The AUDIT was initially developed to detect “hazardous or harmful” alcohol use i.e. cut-off *≥* 8 [4], which represent a broader spectrum of drinking problems than dependence alone. Over time, however, the AUDIT has been increasingly used as a screening tool for AUD, encompassing both terms as alcohol abuse and alcohol dependence. Similar to our approach in the present study, several studies have by now evaluated its performance in identifying individuals who meet the diagnostic criteria for AUD and not just hazardous use [6, 11, 14, 17].

The high Cronbach’s alpha values (0.88) for the AUDIT and AUDIT-C likely reflects strong internal consistency in our sample, but can also indicate other factors, including item redundancy, cultural homogeneity, and high correlations between conceptually related items (e.g., drinking frequency and quantity) [44, 45]. While these factors could play a minor role, the robust psychometric properties of the AUDIT and its consistent performance across studies make high internal consistency the most likely explanation for the observed alpha values.

The AUDIT, AUDIT-C, and Danish national low-risk consumption levels are not systematically used for brief interventions (SBI) or referral to treatment (SBIRT) in Danish hospitals. To our knowledge, no study has investigated current SBIRT procedures across Danish hospitals. Such investigations could shed light on important patient pathways through different SBIRT approaches which might vary markedly depending on factors such as the screening location, timing, referral options or screening tool used, e.g., electronic as in the present study.

## Methodological considerations

Several limitations should be acknowledged. Firstly, we lacked a gold standard. No Danish questionnaire-based or self-reported measure has been validated as gold standard and we did not have access to diagnostic interviews, which would have provided a more robust comparison. We thus relied on self-reported ICD-10 criteria for alcohol dependence which functioned as reference standard [20]. Further, the AUDIT questionnaire does not exist in a formally validated Danish translation, why we relied on the version provided by the Danish Health Authorities [19]. While we acknowledge the limitations of self-reported data, typically regarding underreporting on alcohol consumption [46], self-reported data have shown to offer reliable and valid insights in both national survey estimates [40] and in clinical studies [47, 48] and studies have shown that self-reported questionnaires are generally reliable [49–51].

Secondly, the sample size was small and drawn from a single department as a single-center project, limiting the scope and generalizability of the findings. The convenience sampling approach also introduces a risk of selection bias, which can further limit the generalizability. Additionally, the study population is unlikely to be representative of the general population regarding hazardous drinking and dependence in younger or older age groups, or in ethnically diverse populations. It would be relevant to investigate age dependent and gender-specific cut-off values, especially regarding the AUDIT-C cut-off values, which was not possible in the present study due to the limited distribution of scores among women in our sample.

Thirdly, due to logistical considerations, the study design was cross-sectional, precluding the assessment of changes in alcohol consumption over time and the re-testing reliability of the questionnaires. Lastly, we aimed to validate the AUDIT using a sample size that reflected the variation in alcohol intake based on the estimated prevalence of alcohol problems in the study setting. Consequently, we did not count the total number of patients approached. This may also limit the generalizability of the findings and our ability to report on the sample’s representativeness.

## Conclusion

In a Danish gastroenterology and hepatology department, the AUDIT and AUDIT-C demonstrated poor diagnostic abilities in detecting alcohol problems with certainty but identified patients unlikely to have an alcohol problem with very high certainty. Future studies should investigate the validity of the AUDIT and AUDIT-C cut-off scores in the Danish general population along with gender and age differentiated cut-off scores.

## Electronic supplementary material

Below is the link to the electronic supplementary material.

Supplementary Material 1

Supplementary Material 2

Supplementary Material 3

## Data Availability

No datasets were generated or analysed during the current study.

## Abbreviations

AUC: Area under the curve
AUD: Alcohol Use Disorder
AUDIT-C: The Alcohol Use Disorders Identification Test -Consumption
AUDIT: The Alcohol Use Disorders Identification Test
ICD-10: International Classification of Disease Tenth Revision
NPV: Negative Predictive Value
OPEN: Odense Patient data Explorative Network
PPV: Positive Predictive Value
REDCap: Research Electronic Data Capture
ROC: Receiver Operating Characteristic
SD: Standard Deviation
TLFB: Timeline Follow Back
WHO: World Health Organization
