# Supplementary material for: Validation of the alcohol use disorders identification test in a Danish hospital setting
Source: Subst Abuse Treat Prev Policy. 2025 Feb 14;20:7. doi: 10.1186/s13011-025-00638-w (PMC11829362; doi:10.1186/s13011-025-00638-w)
Supplement: Supplementary file 2 — Supplementary Material 2 [file 13011_2025_638_MOESM2_ESM.docx]

**Supplementary Table 1**. Diagnostic properties of the AUDIT and AUDIT-C scale against ICD-10 ≥ 3 self-reported symptoms of alcohol dependence, for all possible cut-off values of the AUDIT and AUDIT-C scale, N = 300.

|  | AUC | | Sensitivity | | Specificity | | Agreement | |
| --- | --- | --- | --- | --- | --- | --- | --- | --- |
|  | proportion | | % | | % | | % | |
|  | AUDIT | AUDIT-C | AUDIT | AUDIT-C | AUDIT | AUDIT-C | AUDIT | AUDIT-C |
| AUDIT, continuous scale | 0.99 | 0.98 | N/A | N/A | N/A | N/A | N/A | N/A |
| AUDIT, dichotomized according to cut-off value | | | | | | | | |
| ≥ 0 | 0.50 | 0.50 | 100.0 | 100.0 | 0.0 | 0.0 | 4.7 | 4.7 |
| ≥ 1 | 0.60 | 0.61 | 100.0 | 100.0 | 19.6 | 22.4 | 23.3 | 26.0 |
| ≥ 2 | 0.65 | 0.67 | 100.0 | 100.0 | 30.1 | 34.3 | 33.3 | 37.3 |
| ≥ 3 | 0.72 | 0.74 | 100.0 | 100.0 | 43.7 | 47.9 | 46.3 | 50.3 |
| ≥ 4 | 0.81 | 0.84 | 100.0 | 100.0 | 61.5 | 68.5 | 63.3 | 10.0 |
| ≥ 5 | 0.86 | 0.89 | 100.0 | 100.0 | 71.7 | 78.3 | 73.0 | 79.3 |
| **≥ 6** | 0.90 | **0.94** | 100.0 | **100.0** | 79.4 | **87.7** | 80.3 | **88.3** |
| ≥ 7 | 0.93 | 0.91 | 100.0 | 85.7 | 86.7 | 93.4 | 87.3 | 93.0 |
| ≥ 8 | 0.95 | 0.91 | 100.0 | 85.7 | 90.6 | 96.5 | 91.0 | 96.0 |
| ≥ 9 | 0.96 | 0.85 | 100.0 | 71.4 | 92.0 | 98.3 | 92.3 | 97.0 |
| ≥ 10 | 0.97 | 0.78 | 100.0 | 57.1 | 93.4 | 99.3 | 93.7 | 97.3 |
| ≥ 11 | 0.98 | 0.71 | 100.0 | 42.9 | 95.5 | 100.0 | 95.7 | 95.3 |
| ≥ 12 | 0.98 |  | 100.0 |  | 96.5 |  | 96.7 |  |
| **≥ 13** | **0.99** |  | **100.0** |  | **97.2** |  | **97.3** |  |
| ≥ 14 | 0.96 |  | 92.9 |  | 97.6 |  | 97.3 |  |
| ≥ 16 | 0.92 |  | 85.7 |  | 97.9 |  | 97.3 |  |
| ≥ 18 | 0.92 |  | 85.7 |  | 99.0 |  | 98.3 |  |
| ≥ 19 | 0.89 |  | 78.6 |  | 99.0 |  | 98.0 |  |
| ≥ 20 | 0.85 |  | 71.4 |  | 99.3 |  | 98.0 |  |
| ≥ 23 | 0.78 |  | 57.1 |  | 99.7 |  | 97.7 |  |
| ≥ 25 | 0.75 |  | 50.0 |  | 99.7 |  | 97.3 |  |
| ≥ 27 | 0.75 |  | 50.0 |  | 100.0 |  | 97.7 |  |
| ≥ 28 | 0.64 |  | 28.6 |  | 100.0 |  | 96.7 |  |
| ≥ 30 | 0.61 |  | 21.4 |  | 100.0 |  | 96.3 |  |
| ≥ 33 | 0.57 |  | 14.3 |  | 100.0 |  | 96.0 |  |
| ≥ 38 | 0.54 |  | 7.1 |  | 100.0 |  | 95.7 |  |

Notes: ICD-10 ≥ 3 symptoms of alcohol dependence as reference standard for alcohol dependence.
Youden’s index estimates optimal cut-off as AUDIT ≥ 13 and AUDIT-C ≥ 6.
AUDIT, The Alcohol Use Disorder Identification Test. ICD-10, International Classification of Diseases 10^th^ revision. AUC, area under the receiver operator characteristics curve. Agreement, the degree to which the test matches the results of the reference standard (ICD-10 ≥ 3 symptoms of alcohol dependence)

**Supplementary Table 2**. Diagnostic properties of the AUDIT and AUDIT-C scale against Weekly use > 10 drinks/week, for all possible cut-off values of the AUDIT and AUDIT-C scale, N = 300

|  | AUC | | Sensitivity | | Specificity | | Agreement | |
| --- | --- | --- | --- | --- | --- | --- | --- | --- |
|  | proportion | | % | | % | | % | |
|  | AUDIT | AUDIT-C | AUDIT | AUDIT-C | AUDIT | AUDIT-C | AUDIT | AUDIT-C |
| AUDIT, continuous scale | 0.91 | 0.93 | N/A | N/A | N/A | N/A | N/A | N/A |
| AUDIT, dichotomized according to cut-off value | | | | | | | | |
| ≥ 0 | 0.50 | 0.50 | 100.0 | 100.0 | 0.0 | 0.0 | 10.7 | 10.7 |
| ≥ 1 | 0.60 | 0.62 | 100.0 | 100.0 | 20.9 | 23.9 | 29.3 | 32.0 |
| ≥ 2 | 0.66 | 0.68 | 100.0 | 100.0 | 32.1 | 36.6 | 39.3 | 43.3 |
| ≥ 3 | 0.73 | 0.76 | 100.0 | 100.0 | 46.6 | 51.1 | 52.3 | 56.3 |
| **≥ 4** | 0.83 | **0.87** | 100.0 | **100.0** | 65.7 | **73.1** | 69.3 | **76.0** |
| ≥ 5 | 0.81 | 0.85 | 87.5 | 87.5 | 75.0 | 82.1 | 76.3 | 82.7 |
| **≥ 6** | **0.84** | 0.83 | **84.4** | 75.0 | **82.8** | 90.7 | **83.0** | 89.0 |
| ≥ 7 | 0.81 | 0.77 | 71.9 | 59.4 | 89.2 | 95.5 | 87.3 | 91.7 |
| ≥ 8 | 0.74 | 0.69 | 56.3 | 40.6 | 91.4 | 95.6 | 87.7 | 90.7 |
| ≥ 9 | 0.73 | 0.65 | 53.1 | 31.3 | 92.5 | 98.1 | 88.3 | 91.0 |
| ≥ 10 | 0.72 | 0.60 | 50.0 | 21.9 | 93.7 | 98.8 | 89.0 | 90.7 |
| ≥ 11 | 0.68 | 0.56 | 43.8 | 12.5 | 95.2 | 99.3 | 89.7 | 90.0 |
| ≥ 12 | 0.68 |  | 37.5 |  | 95.6 |  | 89.3 |  |
| ≥ 13 | 0.65 |  | 34.4 |  | 95.9 |  | 89.3 |  |
| ≥ 14 | 0.65 |  | 34.4 |  | 96.6 |  | 90.0 |  |
| ≥ 16 | 0.64 |  | 31.3 |  | 97.0 |  | 90.0 |  |
| ≥ 18 | 0.61 |  | 25.0 |  | 97.4 |  | 89.7 |  |
| ≥ 19 | 0.61 |  | 25.0 |  | 97.8 |  | 90.0 |  |
| ≥ 20 | 0.60 |  | 21.9 |  | 98.1 |  | 90.0 |  |
| ≥ 23 | 0.59 |  | 18.8 |  | 98.9 |  | 90.3 |  |
| ≥ 25 | 0.57 |  | 15.6 |  | 98.9 |  | 90.0 |  |
| ≥ 27 | 0.56 |  | 12.5 |  | 98.9 |  | 89.7 |  |
| ≥ 28 | 0.53 |  | 6.3 |  | 99.3 |  | 89.3 |  |
| ≥ 30 | 0.53 |  | 6.3 |  | 99.6 |  | 89.7 |  |
| ≥ 33 | 0.51 |  | 3.1 |  | 99.6 |  | 89.3 |  |
| ≥ 38 | 0.52 |  | 3.1 |  | 100.0 |  | 89.7 |  |

Notes: Weekly use > 10 drinks/week as reference standard for hazardous use.
Youden’s index estimates optimal cut-offs as AUDIT ≥ 6 and AUDIT-C ≥ 4.
AUDIT, The Alcohol Use Disorder Identification Test. ICD-10, International Classification of Diseases 10^th^ revision. AUC, area under the receiver operator characteristics curve.
Agreement, the degree to which the test matches the results of the reference standard (Weekly use > 10 drinks/week)
