# Supplementary material for: Validation of the alcohol use disorders identification test in a Danish hospital setting
Source: Subst Abuse Treat Prev Policy. 2025 Feb 14;20:7. doi: 10.1186/s13011-025-00638-w (PMC11829362; doi:10.1186/s13011-025-00638-w)
Supplement: Supplementary file 3 — Supplementary Material 3 [file 13011_2025_638_MOESM3_ESM.pdf]

ICD-10

Tag udgangspunkt i, hvordan det har været for dig indenfor de sidste 12 måneder.

SPØRGSMÅL

1. Føler du et stærkt ønske om, eller trang til, at drikke alkohol?
   - ○ Ja
   - ○ Nej

2. Når du drikker alkohol, drikker du så ofte mere end du havde i sinde?
   - ○ Ja
   - ○ Nej

3. Har du haft mindre tid til dit arbejde, til dine fritidsinteresser eller til at være sammen med andre, på grund af dit alkoholindtag?
   - ○ Ja
   - ○ Nej

4. Har du drukket mere alkohol for at opnå samme effekt som tidligere?
   - ○ Ja
   - ○ Nej

5. Har du oplevet at du rystede på hænderne, begyndte at svede eller følte dig utilpas efter du reducerede dit alkoholindtag (eller holdt en pause)?
   - ○ Ja
   - ○ Nej

6. Har du oplevet at fortsætte med at drikke, selvom du vidste at alkohol forårsager fysiske eller psykiske problemer?
   - ○ Ja
   - ○ Nej

ANTAL JA = _______
